# Single-Domain Antibodies As Versatile Affinity Reagents for Analytical and Diagnostic Applications

**DOI:** 10.3389/fimmu.2017.00977

**Published:** 2017-08-21

**Authors:** Gualberto Gonzalez-Sapienza, Martín A. Rossotti, Sofía Tabares-da Rosa

**Affiliations:** ^1^Cátedra de Inmunología, Facultad de Química, Instituto de Higiene, UDELAR, Montevideo, Uruguay

**Keywords:** nanobodies, VHH, immunodetection, phage display, imaging, haptens

## Abstract

With just three CDRs in their variable domains, the antigen-binding site of camelid heavy-chain-only antibodies (HcAbs) has a more limited structural diversity than that of conventional antibodies. Even so, this does not seem to limit their specificity and high affinity as HcAbs against a broad range of structurally diverse antigens have been reported. The recombinant form of their variable domain [nanobody (Nb)] has outstanding properties that make Nbs, not just an alternative option to conventional antibodies, but in many cases, these properties allow them to reach analytical or diagnostic performances that cannot be accomplished with conventional antibodies. These attributes include comprehensive representation of the immune specificity in display libraries, easy adaptation to high-throughput screening, exceptional stability, minimal size, and versatility as affinity building block. Here, we critically reviewed each of these properties and highlight their relevance with regard to recent developments in different fields of immunosensing applications.

## Introduction

**Figure 1:**
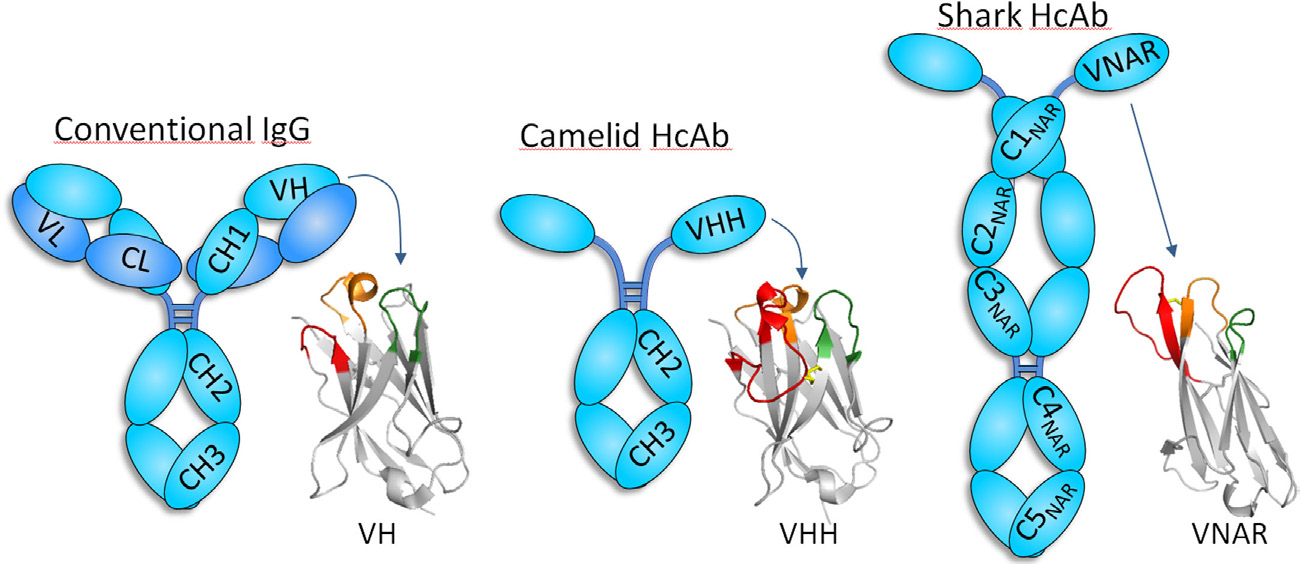
Schematic representation of conventional and heavy-chain-only antibodies (HcAbs). Conventional antibodies, formed by heavy (cyan) and light (blue) chains, are found in all vertebrate, but camelids and cartilaginous fish also have antibodies devoid of light chain, whose antigen-binding site sits exclusively in the heavy chain variable domain. The organization of the heavy chain variable domain of each of these antibodies is shown using three representative structures [PDB IDs: 5WT9 (VH); 5TP3 (VHH); and 2COQ (V-NAR)]. CDR1, CDR2, and CDR3 are depicted in orange, green, and red, respectively, except for V-NAR that lacks CDR2, and the green color is used to denote the HV4 region that sometimes participates in antigen binding. VHHs and V-NARs usually have long CDR3 and non-canonical disulfide bridges (yellow). While the antigen-binding site of conventional antibodies is formed by the combination of the six CDRs of the heavy and light chain, only three and two CDRs are involved in the formation of binding site of camelid and shark HcAb, respectively. Notice that HcAbs do not have the CH1 or an equivalent domain, which forms a strong interaction with the constant domain of the light chain in conventional antibodies.

While most analytical methods rely on the separation of the analyte, the exquisite specificity of antibody recognition allows the detection of trace amounts of the target analyte even in highly complex matrices. This principle of immunodetection was first demonstrated in 1959 when Berson and Yalow developed the first radioimmunoassay for human insulin using a guinea pig antiserum (1). A second major milestone occurred in 1975 when Köhler and Milstein developed the hybridoma technology, enabling the production of high quantities of monoclonal antibodies of the desired specificity (2). Interesting, these two Nobel Prize winning achievements were not patented, which contributed highly to the widespread use of antibodies for immunodetection assays. Further progress in molecular biology and the genetics of antibody diversity added new venues for antibody discovery, with higher control on the selection process and new engineering possibilities (3–5). This paved the way for the current bloom of therapeutic applications of antibodies but also provided the technology to create new assays and biosensors based on the use of recombinant antibody fragments that could be easily tagged and produced at low cost by microbial fermentation (6, 7). All this progress has been dominated by the use of conventional hetero tetrameric antibodies, prototypically represented by the IgG molecule and its fragments (Figure 1), but more recently, the recombinant binding domain of a special type of antibodies devoid of light chain have emerged as a salient alternative for immunosensing. Here, we first present an overview of the heavy-chain-only antibodies (HcAbs) and then outline the characteristics that make them to stand out as unique analytical and diagnostic tools.

## Camelids and Sharks have a Special Type of Antibodies Devoid of Light Chain

**Figure 2:**
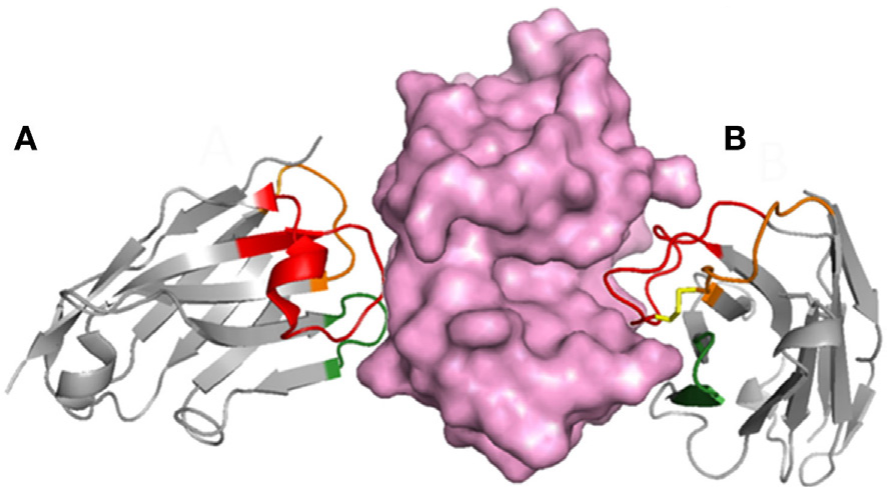
The typically long CDR3 of VHHs provides flexibility to their epitope recognition. The complex of lysozyme (purple) with two VHHs shows that the long CDR3 may contribute to form a flat paratope **(A)** or penetrate in concave epitopes **(B)**; based on PDB structures 1OP9 and 1IR8.

In 1993 researchers from the Vrije University in Brussel reported the existence of a special type of antibodies in camels that were devoid of light chain (8). These, so-called, HcAbs account for up to 50–80% of the circulating antibodies in camels and were also found to be present in the serum of the South American camelids, though in lower concentration (11–25%) (9, 10). Camelid HcAbs have a typical IgG Fc region with dedicated isotypes (IgG2 and IgG3) but lack the CH1 constant domain and have a distinctive variable domain (VHH) with structural features that increase its solubility (Figure 1). Other than camelids, HcAb have not been found in other organisms, with the curious exception of sharks and other cartilaginous fish (Chondrichthyes), the oldest living beings with an adaptive immune system. In addition to heterotetrameric IgM and IgW, these fishes possess the so-called Ig new antigen receptor (IgNAR) (11). IgNARs are formed by two identical heavy chains composed of five constant domains and a dedicated variable domain (V-NAR) (12, 13) (Figure 1). In spite of an evolutionary gap of 425 million years, VHHs and V-NARs share some convergent features that differ from those found in conventional variable domains, more notably, changes in conserved amino acids involved in the VH–VL interaction that make them soluble and independently folding domains, non-canonical Cys pairs in CDRs and frameworks (FRs) that increase their stability and diversity, and higher frequency of hypermutation hotspots and longer than average CDR3 that enlarge their recognition repertoire (11, 14, 15). Formed by fewer CDRs, the antigen-binding sites of VHH and V-NAR domains are smaller than those of conventional antibodies, particularly in V-NARs that present a deletion of the CDR2 region, and thus are formed by 8 instead of 10 β-strands, making them the smallest (12 kDa) antigen-binding domain (16). The reduced paratope and the frequently extended and flexible CDR3 make VHHs and V-NARs particularly capable of binding concave and hidden epitopes (e.g., enzyme active sites, cryptic viral epitopes, etc.) that are not accessible to conventional antibodies (16–19). With no distinctive effector functions associated to their constant domains (20), this unique epitope binding capability has been suggested as the main force that drove the evolution of HcAb (21, 22). Nevertheless, the reactivity of their antigen-binding site is not limited to hidden targets (Figure 2), and HcAbs reacting with a broad range of structurally diverse epitopes have been described, including flat surfaces in macromolecules and small molecules (23–27). Since camelids are easier to handle, produce stronger antibody responses than sharks (28, 29), and the recombinant expression of the heavy chain variable domain of camelid HcAb is typically higher (30), these antibody fragments are more frequently used and will be the focus of this review.

## Camelid HcAbs Bear Independent Folding Variable Domains That Include Mostly VHHs but also VHs

**Figure 3:**
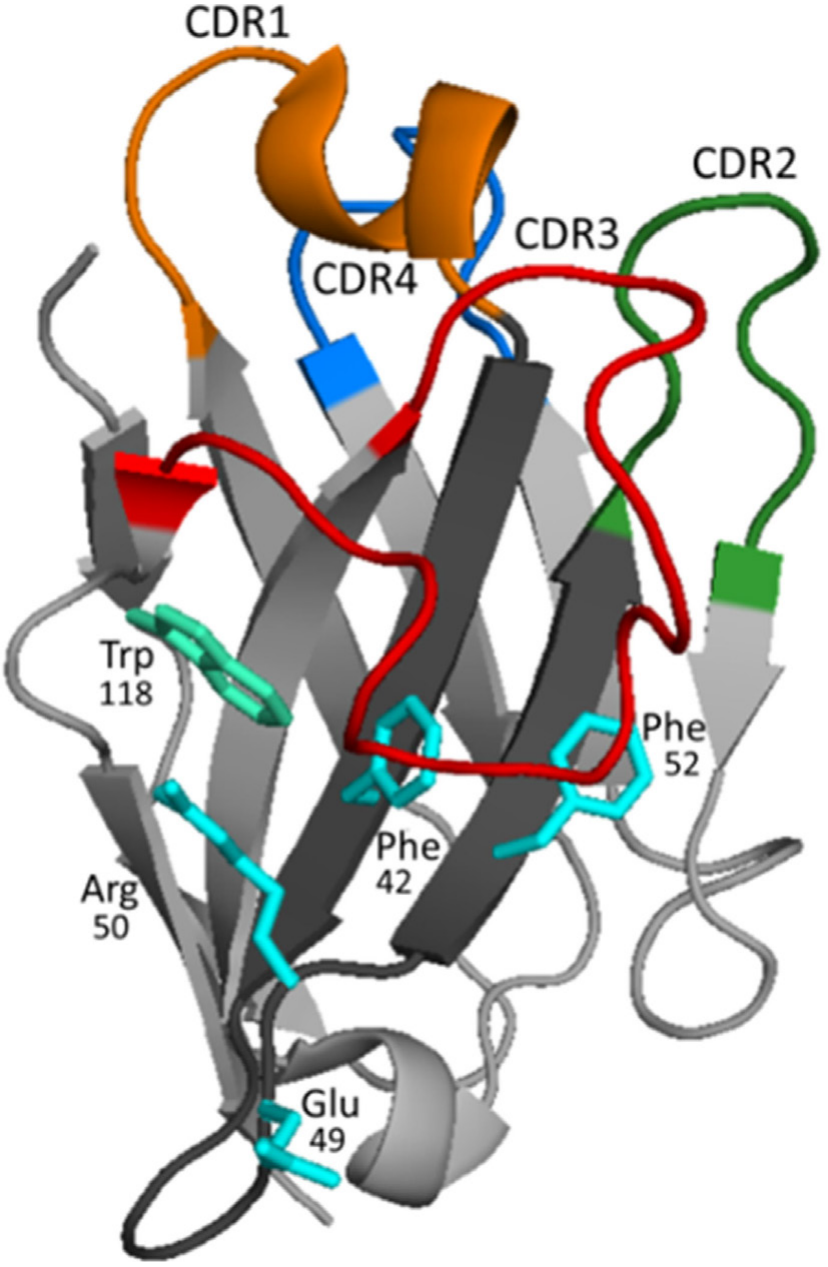
Structure and binding characteristics of the VHH domain. The typical organization of the VHH domain is represented using the structure of a VHH to carbazol (PDB 1U0Q). In framework (FR) 2 (dark gray), the amino acids shown in cyan [in this case Phe42, Glu49, Arg50, and Phe52 (IMGT numbering)] represent the hallmark residues that substitute the critical amino acids of VHs that participate in the interaction with the light chain. CDRs 1, 2, and 3 are colored in orange, green, and red, respectively. The loop of FR 3 shown in blue (also known as CDR4) presents significant variability (higher than in VHs of conventional antibodies) and may also interact with the antigen (41–43). Frequently, the CDR3 is long and bends over FR 2 shielding its hydrophobic residues and helping to mask Trp118 (cyan–green), which is key for the interaction of VH with the VL chain. The structure of the long CDR3s, much more frequently in camels, may be stabilized by non-canonical disulfide bridge, typically formed between Cys residues of CDR1 and CDR3.

The solubility and independent folding of the VHH domain is to a great extent the result of the substitution of conserved residues of FR 2 involved in the VH/VL interaction and the shielding created by the bending of CDR3, Figure 3. In VHHs, Phe/Tyr42; Glu/Gln49; Arg50; and Phe/Gly/Leu52 frequently substitute for the more hydrophobic residues, Val42; Gly49; Leu50; and Trp52 of conventional VH domains, respectively (31). These hallmark substitutions are encoded by dedicated VHH gene segments that are intermixed with the classical VH gene segments in the IgH loci, sharing the same D and J cluster (32). However, not all the variable domains found in HcAb are VHHs. Up to 10% of HcAbs carry conventional VH variable domains (31). The solubility of these VHs is often sustained by a long CDR3 that compensates for the interactions lost by the absence of the light chain, or by the introduction of a hydrophilic residue that substitutes Trp118, which is essential for the interaction with the light chain. This change is often caused by an Arg codon that arises as a consequence of an unusual D–J recombination (33). The camelid HcAb ontogeny is still unclear, but it has been suggested that after recombination of the heavy chain locus in the pre-B cells stage, the poor interaction of the newly formed VHH or “soluble” VH with the VpreB/λ5 surrogate light chain precludes the release of the IgM heavy chain that is bound through the unfolded CH1 domain to the BiP protein in the endoplasmic reticulum (34). This arrests B cell development, but in camelids it might trigger a class switch to the IgG2 and IgG3 constant genes, which resumes cell development because in these isotypes the CH1 region is lost by defective splicing of the RNA primary transcript and thus, the HcAb BCR can be exported to the cell surface (35, 36). This is supported by the fact that light-chain-deficient (L^−/−^) mice and L^−/−^ chicken spontaneously produce HcAbs, but only after deletion of the CH1 domain due to an imprecise recombination event at the genomic level (37, 38). This mechanism selects for independently folding soluble domains, which has important practical consequences, because they can be expressed as stable recombinant fragments with outstanding biotechnological properties. The terms VHH antibody, single-domain antibody, or nanobody (Nb) are often used to refer to the recombinant form of these fragments, but the former can be confusing because, as we have seen, they sometimes consist of soluble VH fragments. For instance, in different studies we have selected both VHHs and VHs against human soluble epoxy hydrolase and tetanus toxin. In both cases, the recombinant VHs and VHHs had similar affinity (in the nanomolar to subnanomolar range) and showed also similar levels of expression and stability (39, 40). In the following sections, we will highlight the properties of Nbs that make them a salient option for immunosensing applications.

## Nbs Can be Easily Selected from Display Libraries because the Immune Specificity is Fully Recovered

**Figure 4:**
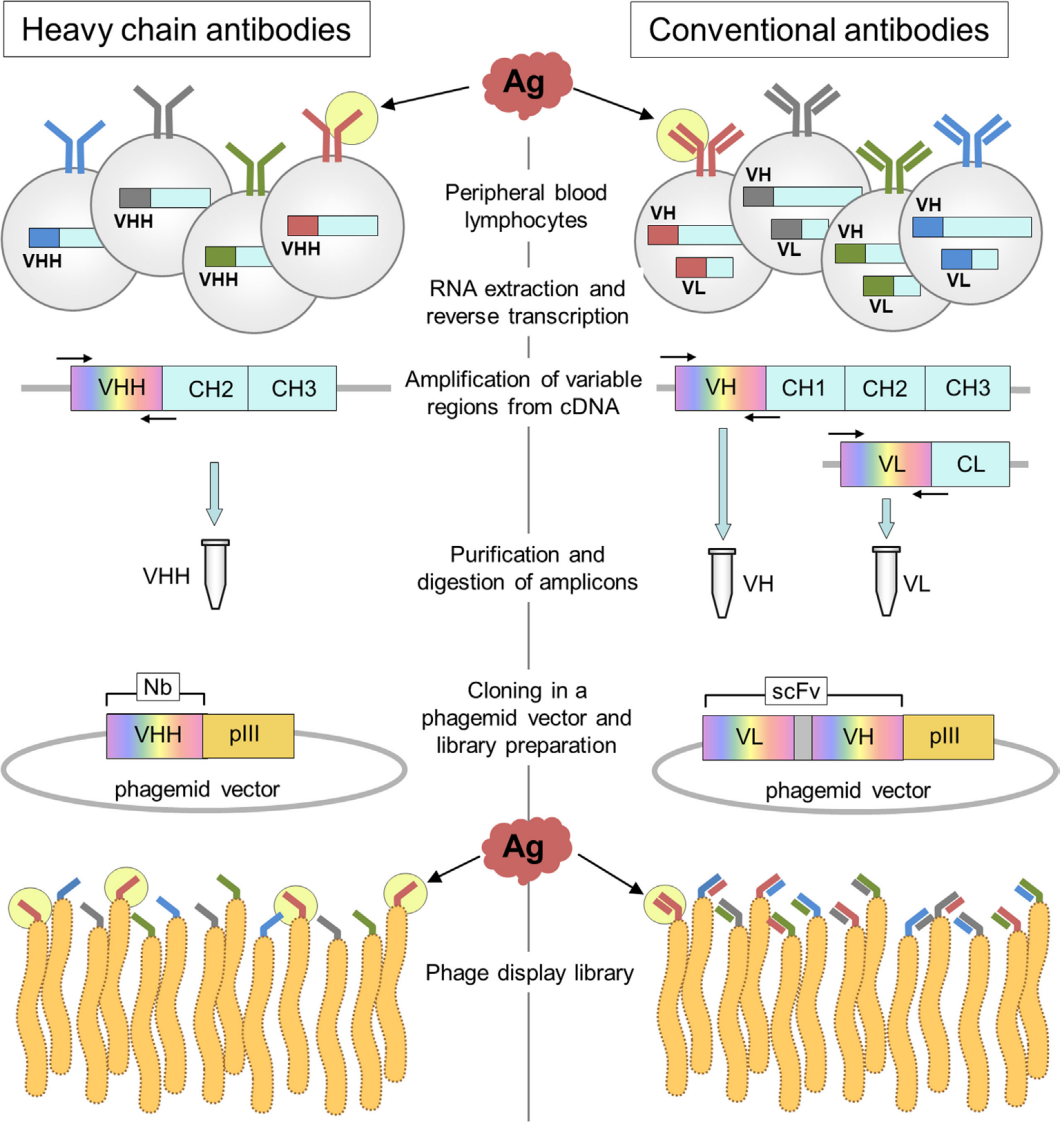
Schematic representation of the construction of heavy-chain-only antibody and conventional antibody libraries. The main steps in the generation of nanobody (Nb) and conventional antibody (scFv) libraries are outlined on the left and right, respectively. The key difference occurs during PCR amplification of the variable gene regions that is done separately for the VH and VL genes. Upon assembly of the VL spacer–VH cassette that encodes the scFv, the VL and VH genes are shuffled, and the original pairing of the light and heavy variable domains occurs only at a very low frequency.

Dromedaries, llamas, and alpacas are not the easiest animals to breed, and their immunizations require larger antigen doses than smaller animals; however, the generation of HcAb libraries stands out as a highly convenient option to easily generate specific binders, because the immune specificity is not shuffled during the construction of the library, Figure 4. In practical terms, that means that the size of HcAb antibody libraries can be several orders smaller than that of conventional antibody libraries to reach a similar representation of the original immune repertoire. The magnitude of this matter can be inferred from the data provided by next-generation sequencing (NGS) of VHH libraries. Recently, the specific repertoire of an immune llama VHH library of 4.8 × 10^8^ independent transformants was studied by data mining of NGS data. Using a sequence identity threshold defined by cluster analysis of the publicly available VHH sequences, the authors were able to identify a wealth of up to >5,000 potential antigen-binding sequences, 90% of which were confirmed as actual functional binders (44). However, many of these binders are not necessarily highly represented in the library. The NGS analysis of the cDNA obtained from a llama immunized with staphylococcal enterotoxin B revealed an exceptional VHH diversity, because out of 5.4 million intact sequences, 88% (4.3 million) were present at a single copy number (45). Just in this case, a VHH library of 5 × 10^6^ transformants would provide a good representation of this diversity, while in theory due to the VH/VL shuffling, an scFv library of (5 × 10^6^)^2^ = 2.5 × 10^13^ transformants would be required to have a similar coverage of the original specificity repertoire. In our experience, when the immune antibody titer is >1/2,000, even a modest size VHH library of 10^6^ individual transformants will easily yield high-affinity binders after one or two rounds of panning, even if a rare epitope-restricted selection is performed (36). Excellent reviews exist on the preparation of single-domain antibody libraries, for instance, see Pardon et al. (46).

## The Simple Nature of Nbs Makes Them Particularly Amenable to High-Throughput Screening

While direct screening of the enriched phages after panning is generally satisfactory for initial selection of binders, the identification of antibodies with the desired diagnostic or analytical properties requires a more careful screening. This is facilitated by transference *en masse* of the enriched VHHs into a strong expression vector generating different antibody constructs that can be interrogated in a flexible way. A particularly useful modification is the metabolic addition of biotin to the C-terminal peptide tag GLNDIFEAQKIEWHE. In the context of this peptide the side chain of its Lys residue is coupled with biotin by overexpression of the biotin ligase BirA of *Escherichia coli* during the production of the Nb (47). We recently adopted this strategy to develop a method for high-throughput screening of Nbs to cell receptors (48). The *in vivo* biotinylation facilitates the parallel analysis of hundreds of Nb clones by cell cytometry, because there is no need of blocking the interactions of the primary or secondary antibodies in case of cells expressing Fc receptors. The strong biotin–streptavidin binding also allows for stringent conditions during pull-down experiments yielding neat finger printings for mass spectrometry identification. Moreover, by adding or not adding biotin during the expression of the Nbs, each clone can be produced with or without this label, which allows to perform competitive epitope binning of the selected Nbs directly on the cells (48). Once selected, the biotinylated Nbs are ready-to-use reagents for cell cytometry diagnosis or immunohistochemistry (49). The method was also adapted to screen for the best Nb pairs to develop two-site immunoassays. After panning, the biotinylated Nbs are produced in 96-well culture blocks and after biotin separation using 96-well Ni-NTA agarose column plates, the eluted Nbs are used to saturate the binding capacity of avidin-coated wells. In this way, equal amounts of the oriented antibodies are retained in each well that can next be probed with an optimized amount of the labeled antigen to spot the clones producing the highest readouts. Once the best capture antibodies are selected, they can be tested in parallel against the rest of the Nb clones using trace amounts of the antigen in a sandwich format. Using this method, we empirically found a pair of Nbs that performed with a detection limit of 63 pg/mL of human soluble epoxy hydrolase in highly complex matrices (40). The use of biotinylated Nbs for two-site immunoassay also has the advantage of the uniform orientation of the immobilized antibody, which has been show to contribute to enhance the assay sensitivity for the detection of bacterial toxins and influenza virus (50, 51).

## Nbs are Stable Under Chemical and Physicochemical Stringent Conditions

Robustness is a desirable property of any antibody, but for some uses, it may be critical. This is the case of analytical applications that are performed under stringent conditions (i.e., high solvent concentration, high temperature, etc.). A striking example is an anti-caffeine llama Nb that allowed determination of the alkaloid in beverages as hot as 70°C (52). Similarly a VHH to a red azo dye was shown to be active in a binding assay at 90°C (53). The thermal stability of Nbs also translates into prolonged shelf lives. For instance, the unaltered storage of microelectrodes intended for point of care detection of ricin increased 7-fold when conventional antibodies were substituted by single-domain antibodies (54). Nbs possess high conformational stability with native-to-unfold free energy transitions between 30 and 60 kJ mol^−1^, and are generally resistant to thermally induced denaturation with melting temperatures in the 60–80°C range (55, 56). By random mutagenesis and stringent selection, their thermal stability can be further improved as shown by Turner et al. that selected a double mutant of an anti-SEB toxin with a melting point of 90°C that was 6.5°C higher that the parent antibody. However, their distinctive property is the reversibility of the thermal unfolding process that allows some VHHs to regain functionality at room temperature even after 1–2 h at extreme temperatures (57). This unfolding–refolding process occurs through a simple two-state mechanism, without intermediate states that would lead to aggregation (58). However, depending on their sequence, not all VHH are able to return to the native conformation. In some cases, they will tend to aggregate when in the unfolded state due to lack of sufficient charge repulsion. This was evidenced studying the thermal stability of Nbs to ricin, which showed a significant improvement in their refolding properties after the introduction of additional charged residues by mutations or addition of fusion tails (59, 60). Aggregation can also occur due to the chemical modifications caused by heat treatment (e.g., Asn deamidation), which could lead to irreversible denaturation. In these cases, the mutation of Asn residues to Asp has also shown to increase the heat resistance of the Nb (61). Likewise, the removal of the extra disulfide bonds has proved beneficial for the thermal resistance of Nbs, although this modification has a negative impact in their thermodynamic stability (62).

Nanobodies have also been shown to tolerate the presence of organic solvents, which are frequently needed for analyte extraction. High tolerance to solvents avoids dilution of the sample with the corresponding gain in sensitivity. For example, efficient extraction of gliadins for the analysis of gluten-free products requires the use of solvents as well as reducing and denaturing agents that were shown to interfere with the binding of conventional antibodies causing false negative results. Careful selection from a VHH library allowed the development of an Nb-based immunoassay that detected gliadins in the presence of 15% ethanol, 0.5% 2-mercaptoethanol, and 0.5 M guanidine hydrochloride (63). Similarly, He et al. found that two of their anti-aflatoxin B1 Nbs were still reactive in the presence of up to 80% of methanol, 80% acetone, or 20% acetonitrile, which allowed them to develop an immunoassay for aflatoxin B1 that could be used for direct analysis of 70% methanol extracts of the toxin (64). We found similar results with Nbs against the pyrethroid metabolite 3-phenoxybenzoic acid. While the best Nb immunoassay was unaffected by the presence of 50% methanol or 50% DMSO, an equivalent polyclonal antibody assay rapidly lost activity. Interestingly, the addition of methanol even improved the assay sensitivity (65).

## When Being Small Matters

The small size of Nbs is in itself an advantage in different analytical and diagnostic applications. One of the clearest examples is their use for *in vivo* molecular imaging (66, 67). Conventional monoclonal antibodies conjugated to different probes have been used for tumor imaging, but their large size (150 kDa) impairs tumor penetration and after administration, they circulate in the blood for several days, requiring long waits and making it difficult to achieve high-contrast images. In addition, their Fc region can cause inappropriate activation of immune effector mechanisms leading to toxic effects. On the other hand, the small size (15 kDa) of Nbs enables a rapid biodistribution and homogeneous tumor labeling, whereas the unbound fraction is rapidly cleared by renal filtration and (68–70). Thus, high-contrast images are generated by single-photon emission computed tomography (SPECT), positron emission tomography (PET), or near-infrared imaging as early as 1 h after administration, which contrasts markedly with the time required for imaging with conventional antibodies. For instance, only 45 min were necessary for SPECT imaging of small xenograft tumors with an anti-epidermal growth factor receptor (EGFR) Nb labeled with ^99m^Tc, while at least 24 h were necessary to visualize similar tumors with the IgG1 cetuximab labeled with the same radionuclide (71). The short time require for imaging with Nbs enables the use to short-lived radionuclides, such as ^68^Ga (half-life = 68 min) or ^18^F (half-life = 109.8 min). An anti-EGFR Nb labeled with ^68^Ga allowed PET imaging of xenograft tumors after 1 h postinjection (72). This rapid clearance has a significant impact in the reduction of the patient’s exposure to radiation. Unfortunately, kidney retention due to non-specific reabsorption in the proximal tubuli is a common problem associated with small radiolabeled agents. However, some studies have shown that modification of the polarity of the Nb C-terminal region and co-infusion with gelofusine and positively charged amino acids can contribute to reduce this effect (73). Nbs have also proven to be advantageous targeting contrast agents for optical molecular imaging, which is more cost-effective than other imaging methods. For example, an Nb to EGFR labeled with the near-infrared fluorophore IRDye800CW produced a clear delineation of tumor xenografts in mice, just 30–120 min after administration, while cetuximab labeled with the same fluorophore reached a less homogeneous distribution in the tumor stroma and required longer time for accumulation and clearance (74). Similarly, a VHH against the epidermal growth factor receptor 2 (HER2) was ~20 times faster and yielded a clearer images than trastuzumab for optical detection of human breast tumor xenografts (75). These imaging techniques have been also applied to monitor inflammatory diseases, such as arteriosclerosis and arthritis and to visualize the antitumor immune response (76–79). The methods used to conjugate the Nbs to the imaging probes will be discussed in the following section.

In biosensor, the interaction of the biological recognition component with the target analyte is converted into an electrical or optical signal, because it changes the physicochemical properties of the sensor surface. As we have seen, the great stability of Nbs adds to a better performance and extend the shelf-life of biosensors (54), but their small size (4 nm × 2.5 nm × 3 nm) also contributes to obtaining higher coating density on the sensor surface, which, together with their oriented immobilization has been shown to improve the biosensors performance (80). Controlled immobilization of Nbs onto biosensor surfaces have been accomplished by C-terminal alkyne function *via* Expressed Protein Ligation (81) (see below) and *in vivo* biotinylated Nbs bound to streptavidin (50). Also, the compact size of Nbs enabled their direct use as templates to synthesis highly derivatized gold nanoparticles that were incorporated into microelectrodes and used to detect the analyte by changes on the tunneling current of the gold-antibody networks. Using Nbs and IgG against cholera toxin β-subunit, the gold nanoparticles, templated and formed with both types of antibodies, showed functional recognition of the toxin. However, while the close packing of the Nb nanoparticles made it possible to detect the very low concentrations of the toxin in the 5–50 pg/mL range, negligible signals were obtained with the IgG particles (82).

The reduced size of Nbs proved to be an asset for the development of homogeneous immunoassays base on Förster resonance energy transfer (FRET). Indeed, the efficiency of FRET is highly dependent on the distance between the donor and acceptor fluorophores and rapidly vanishes when the gap is larger than 10 nm. This is the case of sandwich assays set up with whole IgG molecules; however, using a pair of Nbs to EGFR conjugated to a terbium cryptate (Lumi4-Tb) and quantum dots, it was possible to develop a mix-and measure time-gate FRET assay for this biomarker that detected, after 1 h incubation, diagnostic relevant concentrations of circulating EGFR in biological fluids (83). In another study, surface residues selected based on the crystallographic structure of an Nb against the nuclear pore complex (NPC), were mutated to cysteine and used to conjugate fluorescent dyes close to the antigen-binding site. In this way, they produced imaging reagents that could position fluorophores as close as 1–2 nm of NPC targets, which allowed to obtain super resolution images of the NPC using STORM imaging techniques (84).

**Figure 5:**
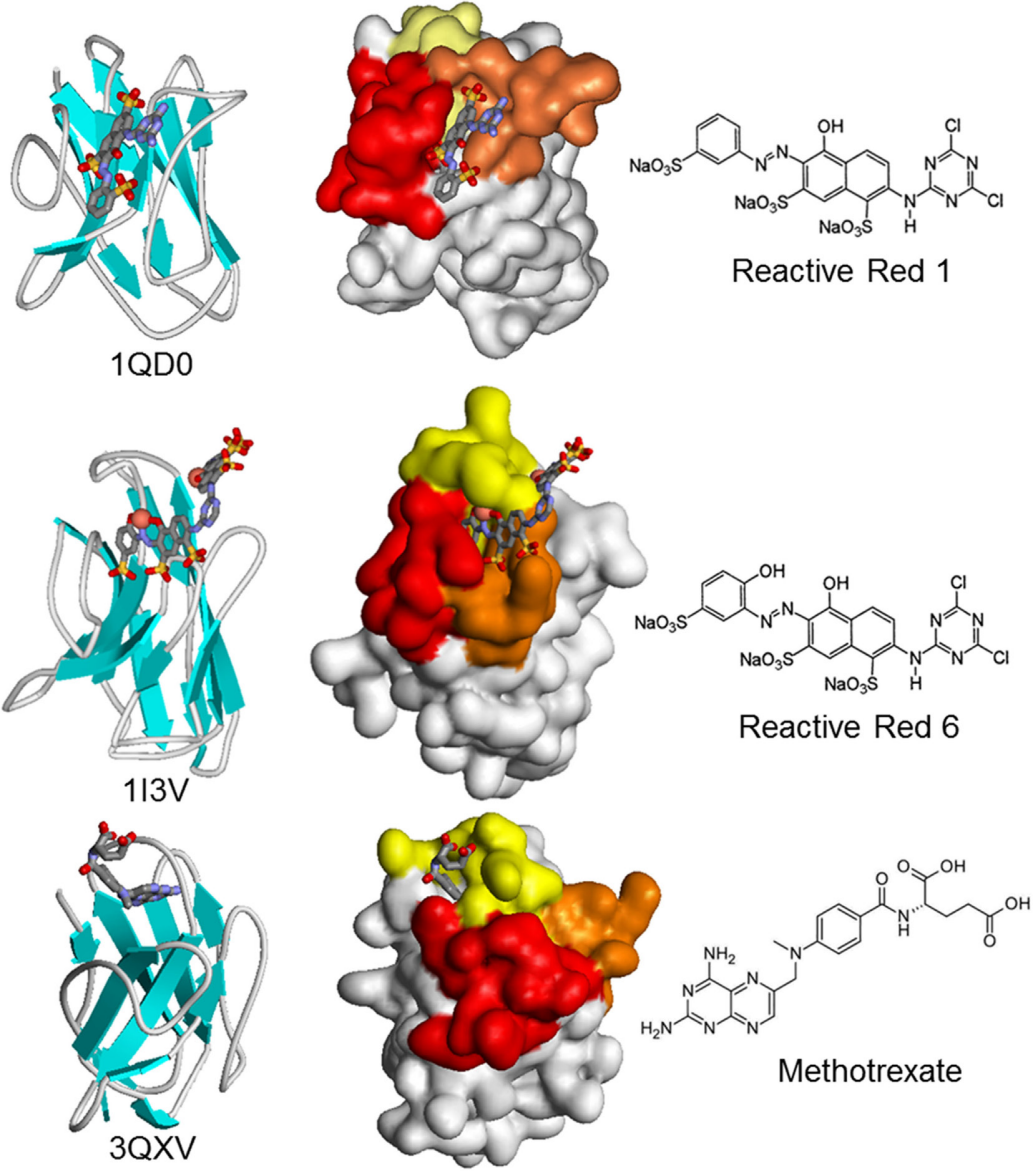
Schematic and surface representation of three VHH structures in complex with haptens (CPK). In the surface representation, CDR1, CDR2, and CDR3 are shown in yellow, orange, and red, respectively. Upon crystallization, two molecules of Reactive Red 6 reacted through the triazine group to form a dimer of the dye.

The minimal size of Nbs may not always be an advantage. This is the case for the isolation of Nbs against small compounds, which are a huge and relevant group of analytes of toxicological, medical, and environmental analytical interest. While the smaller number of CDRs does not seem to threaten the ability of Nbs to bind macromolecular antigens with high affinity, the lack of a light chain may hamper their ability to bind small molecules (haptens). Indeed, conventional antibodies typically bind haptens at the interface of the VH–VL domains (42), and different studies have consistently found that the camelid immune response against small molecules is dominated by conventional IgG1, with lower titers of the IgG2 and IgG3 monodomain isotypes (57, 85, 86). There is very little information on the way in which Nbs bind small molecules, with only three hapten–VHH structures available, corresponding to the azo dyes Reactive Red 1 (733 Da) and Reactive Red 6 (717 Da), and the chemotherapy agent methotrexate (454 Da) (42, 87, 88). There is a great deal of variation in the combining site (Figure 5). Reactive Red 1 accommodates in a lateral fashion in a groove formed between CDRs 2 and 3. Reactive Red 6 binds in a cavity formed by the three CDRs, while the cupper atoms of the hapten interact with two histidines of CDR1. Interestingly, methotrexate is bound in a non-canonical way, being deeply buried in the structure of the Nb in a “tunnel” roofed by CDR1 and closed by residues in a loop of FR3 that the authors identified as CDR4, which participates in direct and critical interactions with the hapten. Indeed, the absence of some residues of this loop causes a loss of up to 1,000-fold in the binding affinity. Surprisingly, the VHH–hapten complexes present a total change in solvent accessible surface area upon binding that is larger than that observed for conventional antibody–hapten complexes, particularly for methotrexate that buries 895 Å^2^ (42).

Despite the unfavorable HcAb immune response against haptens, high-affinity Nbs to a diverse group of small compounds have been generated and used to develop highly sensitive immunoassays (low ng/mL to pg/mL range). Those compounds include the toxins 15-acetyldeoxynivalenol (338 Da), ochratoxin A (404 Da), and aflatoxin B1 (312 Da); the drug methotrexate (454 Da); and the environmental contaminants triclocarban (316 Da), 3-phenoxybenzoic acid (214 Da), tetrabromobisphenol A (543 Da), and brominated diphenyl ether-47 (486 Da), reviewed by Bever et al. (89). In all cases, these Nbs were selected from immune phage display libraries by competitive selection with the free analyte. Recently, using the cyanotoxin microcystin-LR (995 Da) as a model hapten and a high-throughput screening, we compared this way of selection with two additional strategies. We found that selecting for Nbs with the slowest *k*_off_ for the immobilized hapten allowed us to attain a detection limit of 50 pg/mL, which was 10-fold better that the one obtained using the Nbs isolated by competitive selection (90). While the usefulness of this approach still needs to be demonstrated for other haptens, alternative methods for the selection of anti-hapten Nbs are needed, because, conversely to what happens with other antigens, failures in the generation of Nbs that recognize small molecules in solution are common (89). The reasons underlining these failures are unknown, but additional structural studies of small analyte–Nb complexes will help to understand this interaction and allow a more rational design of immunizing and selecting haptens.

## Nbs as Multipurpose Affinity Building Domains

For many analytical and diagnostics applications Nbs have been directly used as phage borne antibody fragments. The M13 phage particle, commonly used for phage display, has ~2,700 copies of the pVIII protein providing a large surface for tracer attachment, which translates in higher assay sensitivity that can be up to two orders of magnitude better than that obtained with the soluble Nb (91). Likewise, the phage DNA can be used for ultrasensitive detection of the phage borne antibody using real-time PCR in different immunoassay formats, which also extends the dynamic range of the assay in several orders of magnitude (92, 93). Nevertheless, being the smallest antibody fragments, VHHs are ideal candidates for building chimeric recombinant proteins for analytical and diagnostic applications. Nbs have been produce as recombinant fusion proteins with hyperactive mutated versions of the alkaline phosphatase (AP) of *E. coli* (94). The combination of target binding with signal transduction domains reduces the number of steps in immunoassay applications (95), and due to the spontaneous dimerization of AP the functional affinity of the Nb partner increases significantly, improving the assay sensitivity. Surface plasmon resonance measurement of the interaction of five VHHs to different bacterial toxins showed in all cases that the *K*_D_ of the VHH–AP fusion was roughly an order of magnitude lower than that of the parent Nb (96). Nbs have also been produced with good yields as bioconjugates of the biotin binding protein rhizavidin and employed as ready-to-use reagents in sandwich immunoassays (97). In another study, Nbs have been fused to bacterial cellulose-binding domains (CBD) for paper based analytical applications. The pentamerizing subunit of verotoxin B was used as scaffold to produce pentavalent constructs and was sandwiched between an anti-*Staphylococcus aureus* VHH and the CBD. The bispecific pentavalent recombinant construct was anchored to a cellulose filter surface through the interaction of the CBD domains providing high avidity for the detection of the bacteria in a flow-through device (98). In an interesting proof of concept, an Nb to red fluorescent protein was expressed as a fusion protein with the magnetosome protein MamC in the magnetite-synthesizing bacterium *Magnetospirillum gryphiswaldense*. This generated Nb decorated biogenic magnetic bioparticles that were ready to be used *in vitro* to pull-down antigens from complex cell extracts (99). Genetic fusion of VHHs to fluorescent proteins, termed chromobodies, have also been devised for live cell microscopy. In a first demonstration, Rothbauer et al. fused and anti-green fluorescent protein (GFP) VHH with the monomeric fluorescent red protein and showed that the chimeric recombinant Nb could detect and track different cell proteins fused to GFP in different cell compartments (100). Recently, zebrafish lines expressing chromobodies were generated and used to captured full localization dynamics of the endogenous targets in the live animal (101).

**Figure 6:**
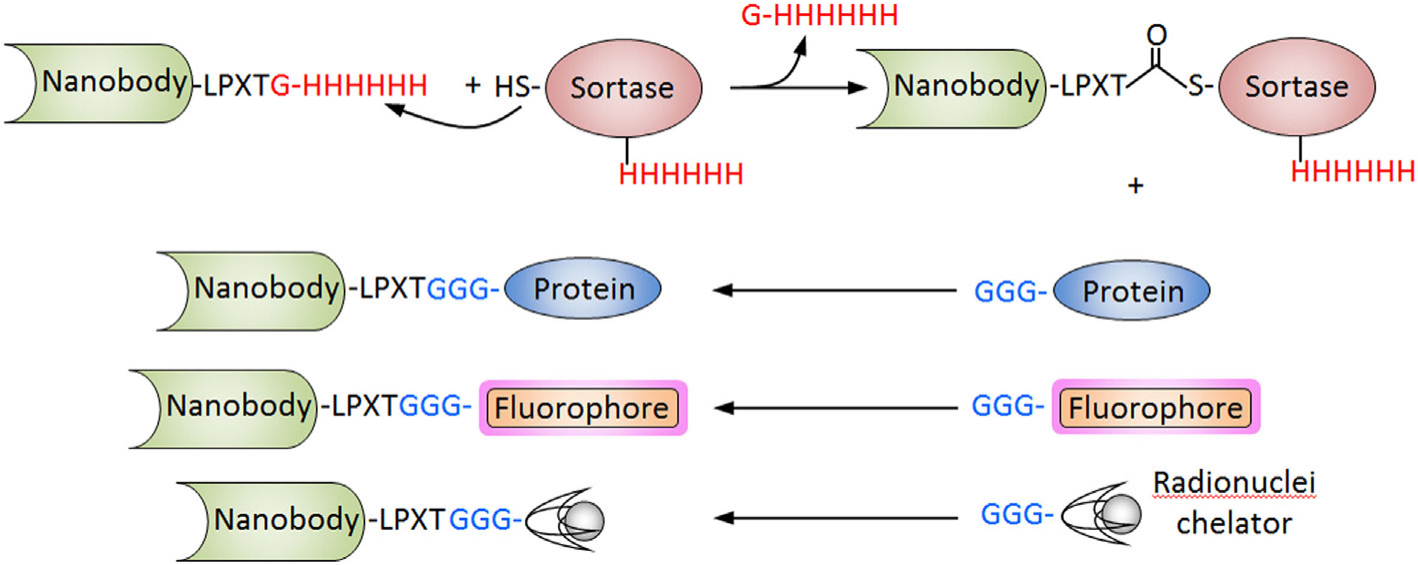
Sortase-mediated conjugation of nanobodies (Nbs). The procedure is schematized for the *Staphylococcus aureus* sortase. The Nb is produced with the C-terminal tag (LPXTG) and is incubated with the recombinant sortase that cleaves the Thr–Gly bond forming an acyl intermediate. The nucleophilic attack of the poly-Gly stretch of the partner protein, fluorophore of chelating compound breaks the thioester forming conjugate. The hexa His tag in the Nb and sortase is used to separate the unreacted Nb and the sortase from the final reaction.

Nanobodies have also been conjugated to fluorophores and radionuclides for immunosensing applications using different strategies that allow site-specific derivatization. We have already discussed the *in vivo* biotinylation of Nbs as a versatile strategy that facilitates their high-throughput screening but also their upstream applications. The addition of a cysteine residue at the carboxyl-terminus has also been used to prepared Nb conjugates. The paired cysteine promotes the spontaneous dimerization of the Nb, but this disulfide bridge can be selective reduced using 2-mercaptoethylamine, allowing the conjugation to maleimide probes with preservation of the VHH functionality (102). A particularly powerful approach is the site-specific sortase-mediated ligation. In this approach, the Nb is expressed with a C-terminal sequence corresponding to the recognition motif of the sortase, commonly -LPXTG for *S. aureus* sortase A or -LPXTA for *Streptococcus pyogenes* sortase A. After incubation with the enzyme, the C-terminal residue is cleaved and a thioester Nb-sortase intermediate is formed, which is resolved by a nucleophilic attack of a short poly-glycine sequence of a protein or peptide conjugate (103) (Figure 6). This strategy was used to conjugate an anti-HER2 Nb to three different imaging probes, the chelating compounds of ^111^In and ^68^Ga for SPECT and PET, respectively, and the fluorescent dye Cy5 for optical molecular imaging. All three conjugates were fully functional and facilitated the attainment of high-contrast images of xenografted tumors after 1 h incubation (104). Sortase has also been used to label Nbs to Mac1 (CD11b/CD18) and class II MHC with ^18^F and ^64^Cu for PET studies. Since these markers are highly expressed in myeloid cells, which are recruited to the tumor microenvironment as part of the immune response, these antibodies allowed clear visualization of the tumors with remarkable specificity, in both, xenogeneic and syngeneic mouse models. Since the course of the antitumor immune response would be affected by therapy, the method may provide additional valuable information for the evaluation of the treatment (78). Sortase-mediated reactions were also used for direct attachment of Nbs in a site-specific manner to graphene oxide nanosheets for the rapid analysis of cell populations. To this end, the graphene oxide surface was initially derivatized with diaminopolyethylene glycol, which was reacted with dibenzocyclooctyne-*N*-hydroxysuccinimidyl ester to make it “click chemistry-ready.” On the other hand, a sortase-mediated reaction was used to conjugate the Nbs against different leukocyte subpopulations to a Gly_3_ peptide equipped with a TAMRA fluorophore and an azide group for the click chemistry reaction. The graphene sheets derivatized with different Nbs were arranged in tandem in a flow chamber, which allowed the characterization of the target cells populations from tiny (30 µL) volumes of blood (105). Intein-mediated protein ligation has also been used to introduce site-specific modifications at the C-terminus of the Nb molecule. Good cytoplasmic expression of an Nb to vascular cell adhesion molecule 1 fused to an intein equipped with a chitin binding domain was obtained in *E. coli* Shuffle T7 cells. The fusion protein was then immobilized onto chitin-agarose and self-cleavage was induced by incubation with cysteine-alkyne, releasing the Nb modified with a clickable alkyne function (106). Using this procedure, biosensors for detecting this atherosclerosis biomarker were generated using a click chemistry reaction that enable the oriented immobilization of the modified Nb onto azide-derivatized silicon baffles or surface plasmon resonance chips (81).

## Conclusion

In the previous sections, we have discussed the distinctive properties of Nbs and recent developments that highlight their enormous potential as immunoreagents. This becomes evident, by the exponential growth in the number of studies reporting their application in innovative immunodetection formats. Nbs have a broad recognition capability and bind their antigens with affinity values that rival those of conventional antibodies. The possibility of comprehensively representing the immune specific repertoire in phage libraries is perhaps the single most outstanding property of the Nb technology. This is particularly needed when not just binding, but epitope specific recognition is required, the case for the differential diagnosis of related pathogens, disease-linked conformation as is determinants, monitoring of chemically similar analytes, etc. In spite of that, some antigens will still require special considerations. As we have seen, new studies will be necessary to improve the rate of success in the preparation of Nbs to small analytes. Similarly, the generation of Nbs against native epitopes on difficult targets, such as cell receptors, may also face some limitations, particularly when recombinant soluble antigens are used. Nbs have a strict conformation-dependent recognition of their epitopes, and the native folding of the antigen can be altered during the preparation of the immunogen, or if direct adsorption onto solid phase is used during panning or screening (46). The existence of dromedary (Dubca ATCC^®^ CRL-2276) and alpaca cell lines (107) that can be used as transfection carriers for immunization, in conjunction with streamlined methods of screening on antigen-expressing cells (48) may offer a solid alternative to overcome these limitations.

The outstanding properties of Nbs will continue to promote innovation, enabling completely novel types of immunodetection developments in research and commercial applications. While the size of the analytical and diagnostic antibody market is only a fraction of the therapeutic antibody business, this is still a multi-billion dollar competitive market. With the recent expiration of the original patents on camelid antibodies and the rapid progress in the technology for their production and selection, we foresee a growing interest of the industry to incorporate Nbs as a new generation of immunoreagents to add a competitive edge to their products, increase robustness, facilitate automation, and produce improved diagnostics.

Finally, Nbs will offer unsurpassed opportunities for standardization. Owing to their simplicity, high yield and straightforward production by bacterial fermentation, once selected and validated for their intended purpose, their ~400 bp DNA sequence will be all that would be required to reproduce their specificity over the years, and in any laboratory in the world. With the continued drop in the cost of synthetic genes, this will also allow the “electronic” exchange of highly standardize binders, avoiding the cost and regulatory inconveniences of shipping hybridomas or limited amounts of protein antibodies. This perfectly fulfills the claims of the scientific community expressed by Bradbury, Plückthun, and 110 cosignatories about the need of advancing toward the use of validated recombinant antibodies that will allow the user community to avoid the frustration and monetary waste caused by the poorly characterized and ill-defined commercial antibodies (108).

## Author Contributions

GG-S—contributed to the conception, design, writing, and revision of the manuscript. MR and ST—contributed to the writing and revision of the manuscript.

## Conflict of Interest Statement

The authors declare that the research was conducted in the absence of any commercial or financial relationships that could be construed as a potential conflict of interest.
